# The Impact of the Spatial Distribution of Ventricular Extrasystoles on Implantable Cardioverter‐Defibrillator Recipients

**DOI:** 10.1111/pace.70033

**Published:** 2025-08-25

**Authors:** Carlos Arthur Hansel Diniz da Costa, Gabriela Menichelli Medeiros Coelho, Rhanniel Theodorus Helhyas Oliveira Shilva Gomes Villar, Gabriela Rodrigues de Oliveira, Pedro Henrique Correia Filgueiras, Enia Lucia Coutinho, Claudio Cirenza, Angelo Amato Vincenzo de Paola

**Affiliations:** ^1^ Federal University of São Paulo São Paulo São Paulo Brazil

**Keywords:** cardiomyopathies, cohort study, implantable cardioverter‐defibrillator, premature ventricular complexes, prognosis

## Abstract

**Introduction:**

Premature ventricular complexes (PVC) are a common phenomenon observed in both normal and pathological heart conditions. However, they do not always behave in the same way. Different PVCs present with varying QRS morphologies, mechanisms, and origin sites. These differences may imply distinct prognoses. To date, the impact of the three‐dimensional distribution of PVCs across the heart on the prognosis of ICD recipients has not been adequately investigated.

**Material and Methods:**

We conducted an ambidirectional cohort study. Patients underwent two twelve‐lead ambulatory ECG recordings during follow‐up. The spatial distribution of PVCs was analyzed using the algorithm proposed by Kuchar et al. The impact of this spatial distribution on clinical variables was assessed using mixed generalized models.

**Results:**

Fifty‐five patients were enrolled, with a mean follow‐up time of 41.12 ± 13.48 months. All patients underwent two 12‐lead ambulatory ECG recordings. The median PVC count was 91.5. PVCs were classified according to the algorithm proposed by Kuchar et al. PVCs arising from exit sites located in the intermediate left ventricle were associated with a higher number of therapies (odds ratio [OR]: 4.78; 95% confidence interval [CI], 1.19–19.26; *p* = 0.028) and prolonged QRS duration. PVCs with exit sites located in the septal region were associated with higher NYHA functional classes (OR: 2.22 [95% CI: 1.08–4.44]; *p* = 0.030). No statistically significant interaction was found between PVC topography and gender, number of ATP episodes, ATP success rate, or number of shock episodes.

**Conclusion:**

The spatial distribution of PVCs influenced the prognosis of ICD recipients.

## Graphical abstract

Distribution of distinct topographies and their relationship with NYHA classes and therapies. Each of the 111 dots represents a distinct PVC topography recorded in the 12L‐AECGs. Black dots represent PVC associated with patients classified as NYHA class I, green dots with NYHA class II, and yellow dots with NYHA class III. Dots with red halos indicate PVCs associated with therapies and the corresponding NYHA class (I or II). The intermedium region of the apicobasal axis is linked to a higher rate of therapies, while the septal region of the lateroseptal axis is associated with higher NYHA classes. A, apical; An, anterior; B, basal; C, central; Im, intermedium; In, inferior; L, lateral; M, medium; S, septal.

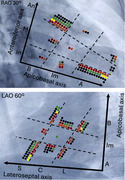

## 1 Introduction

Premature ventricular complexes (PVCs) are common, occurring in up to sixty‐nine percent of ambulatory electrocardiograms [1]. Despite initial concerns about their potential negative prognosis [2], PVCs are generally considered benign and have not been associated with reduced survival rates compared to the general population [3]. Even in adverse conditions, such as ischemic heart disease, PVCs do not correlate with higher mortality rates. On the contrary, efforts to suppress them have led to increased adverse outcomes, as demonstrated in the seminal CAST trial [4].

Since then, PVCs have been regarded as a secondary factor in predicting cardiovascular morbidity and mortality, with the left ventricular ejection fraction taking precedence. This focus has resulted in most PVC studies being conducted in the context of left ventricular dysfunction [5]. However, it is well established that PVCs differ significantly in their manifestation. Their characteristics may vary greatly depending on their underlying mechanisms and origin [6]. The impact of different PVC exit sites on outcomes in implantable cardioverter‐defibrillator (ICD) recipients has yet to be thoroughly evaluated.

## 2 Objective

Evaluate how the three‐dimensional distribution of PVCs across the heart affects the prognosis of ICD recipients.

## 3 Methods

### 3.1 Patient Recruitment

Patients were considered eligible for this study if they were recipients of either an implantable cardioverter‐defibrillator (ICD) or a cardiac resynchronization therapy defibrillator (CRT‐D), and if they were included in our service database. Enrollment occurred when patients returned to the office for regular follow‐up visits. Informed consent was obtained from all participants. This study was approved by the Ethics Committee of the Federal University of São Paulo (CAAE: 64353722.5.0000.5505).

### 3.2 Exclusion Criteria

Patients were deemed ineligible for the study if they presented with acute illness or decompensation at the time of recruitment. Patients were also excluded if they did not attend regular follow‐up appointments. For the purposes of this study, regular follow‐up was defined as a maximum interval of 12 months between evaluations. Patients who did not provide consent were excluded, as were those with fewer than two 12‐lead ambulatory electrocardiograms recorded during follow‐up.

### 3.3 Patient's Follow‐Up

All patients were followed according to our service protocol, which involved an evaluation every 4 months for ICD or CRT recipients. Comorbidities were managed in accordance with current guidelines. Patients were instructed to return earlier in cases of syncope or ICD shocks. Due to the poor socioeconomic background of some patients, follow‐up visits were occasionally delayed to 6 months. This also applied to patients residing in remote or distant locations. The follow‐up period was defined as the time from the first recorded entry in the patient's file to the end of the 12‐month prospective follow‐up.

### 3.4 PVC Origin and Exit Site Definitions

The main determinant of QRS morphology is how the wavefront resulting from the abnormal beat captures the myocardium. In fibrotic substrates, the wavefront must travel through a channel within the scar before capturing the myocardium. Such scars may contain multiple conducting channels. Therefore, a single origin can generate multiple QRS morphologies depending on the channel through which the wavefront exits the scar. For the purposes of this manuscript, we define the origin as the focus from which the abnormal beat arises, and the exit site as the point at which the wavefront captures the myocardium.

### 3.5 Twelve‐Lead Ambulatory Electrocardiograms

The twelve‐lead ambulatory electrocardiogram (12L‐AECG) is an ambulatory electrocardiogram employing the twelve leads of a standard electrocardiogram (Figure S1). In this study, our 12L‐AECGs were reconstructed using seven‐lead recorders (DMS 300–8 system, D.M. Software, Los Angeles, CA, USA) and their proprietary algorithm. Each patient underwent two recordings during the follow‐up period, with a minimum interval of 4 months between recordings. Silver chloride (AgCl) electrodes (Maxicor, Pinhais, BR) were used, and electrode placement was performed by highly trained nurses following the manufacturer's instructions. The sampling rate was 4096 Hz with 8‐bit resolution.

### 3.6 Exams’ analysis and PVC Classification

**FIGURE 1:**
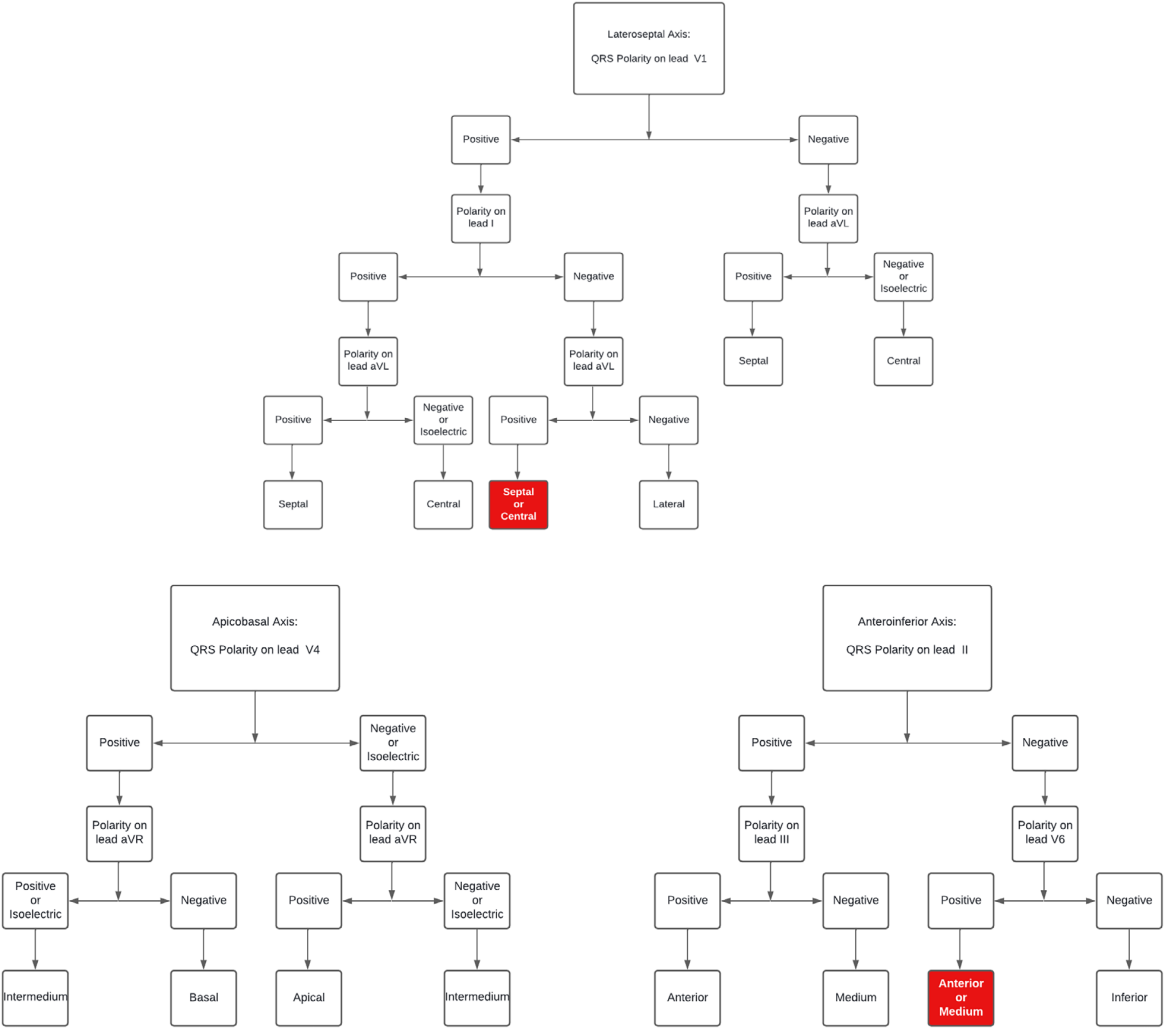
Kuchar's algorithm. The algorithm proposed by Kuchar et al. for classifying ventricular arrhythmias based on QRS polarity of key leads. Red boxes denote topographies that could not be properly classified and were, therefore, excluded from the final analysis. [Colour figure can be viewed at wileyonlinelibrary.com]

All 12L‐AECGs were analyzed by the main author. If the twelve‐lead ambulatory electrocardiograms had been recorded prior to the patient's enrollment in this study, they were reanalyzed by the main author. The spatial distribution of PVCs was classified using the algorithm described by Kuchar et al. [7] (Figure 1). While other algorithms exist for identifying scar‐related PVC exit sites [8, 9], they require the PVC to match a predetermined template or rely on prior knowledge of myocardial infarction topography. Kuchar's algorithm, on the other hand, does not have these limitations, making it more suitable for our population, as Chagas cardiomyopathy was the most prevalent disease among our patients.

**FIGURE 2:**
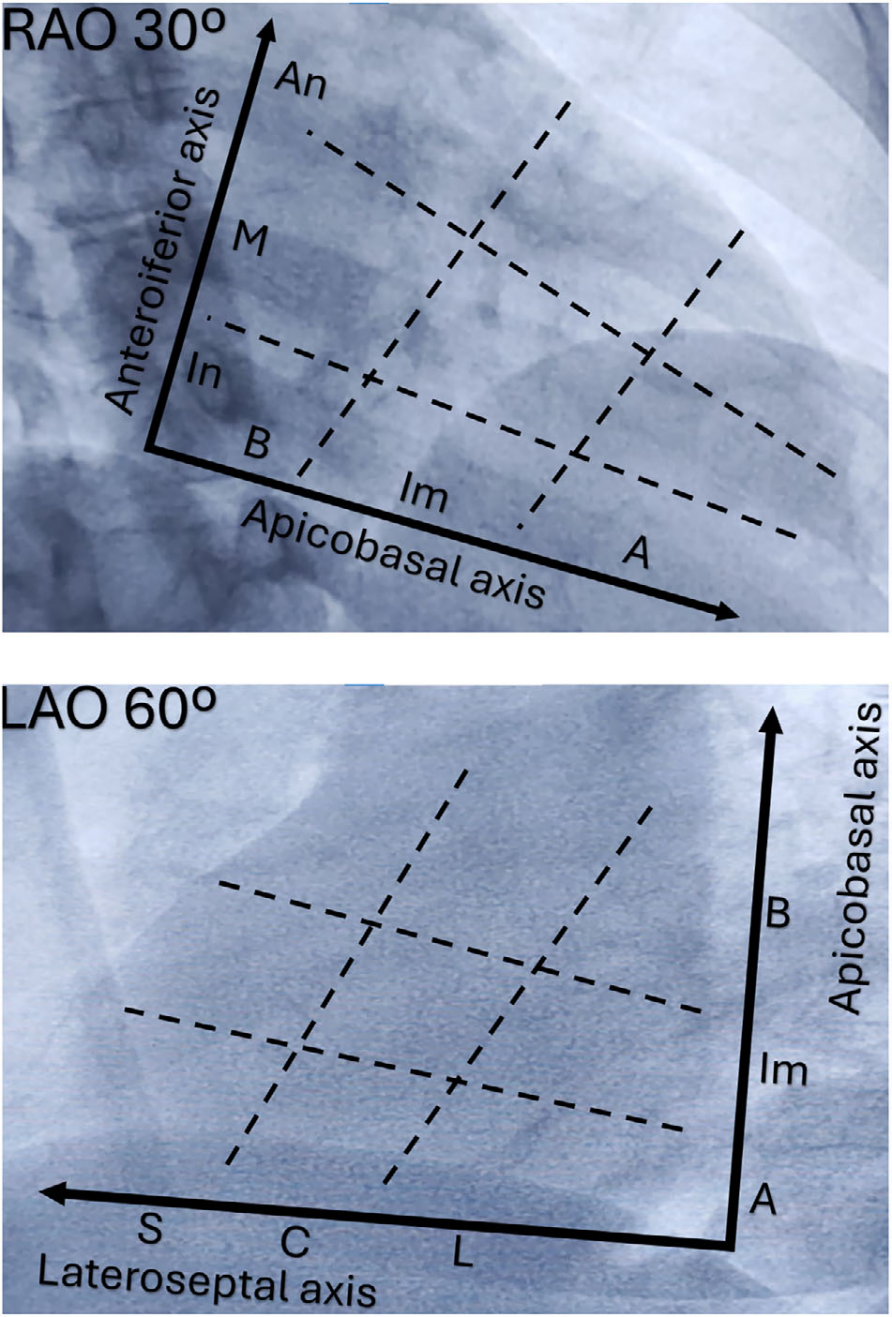
The tridimensional system. The triaxial system. Axes were defined using the right anterior oblique (RAO) and left anterior oblique (LAO) projections. A, apical; An, anterior; B, basal; C, central; Im, intermedium; In, inferior; L, lateral; M, medium; S, septal. [Colour figure can be viewed at wileyonlinelibrary.com]

The algorithm uses a triaxial system: lateroseptal, apicobasal, and anteroinferior, and each one is further divided into three territories (Figure 2). In some cases, the algorithm led to situations where it was not possible to determine the precise axial topography. For instance, a QRS complex that was negative in lead II and positive in V6 could not be properly classified along the anteroinferior axis. When the exit site of a PVC could not be defined, that morphology was excluded from the final analysis.

### 3.7 PVC Axial Topography

The algorithm classified PVCs in three dimensions, corresponding to the three cardiac axes. The axis containing the lateral wall and the interventricular septum was referred to as the lateroseptal axis and was further subdivided into lateral, central, and septal portions. The axis containing the anterior and inferior walls was termed the anteroinferior axis, subdivided into anterior, medium, and inferior portions. Lastly, the axis extending from the base to the apex was called the apicobasal axis, and was divided into basal, intermedium, and apical portions.

The algorithm evaluated each PVC with respect to these three axes, generating three coordinates for each PVC, with each coordinate representing a subdivision of one axis. As an example, a PVC arising from the anterior septum, near the His bundle, would be classified as septal (lateroseptal axis), anterior (anteroinferior axis), and basal (apicobasal axis). These coordinates were referred to as the PVC's axial topographies, and each PVC was assigned three axial topographies.

### 3.8 PVC General Topography

The combination of the three axial topographies defined the PVC general topography. While there were 27 theoretically possible general topographies (3 × 3 × 3), sites where the axial topographies “central” (lateroseptal axis) and “medium” (anteroinferior axis) coexisted were, by definition, mid‐cavity sites. Since it is impossible for a PVC to originate from the mid‐ventricular cavity, there were 24 effectively possible general topographies.

### 3.9 Distinct General Topographies per Patient Definition

We defined distinct general topographies per patient as the number of different PVC general topographies recorded across both 12L‐AECG exams for a single patient. For example, if a patient had two distinct PVC general topographies recorded in the first 12L‐AECG and one in the second, the patient would be assigned two distinct general topographies if the PVC general topography from the second exam matched one of the first exam's morphologies. If it did not match any, the patient would be assigned three distinct general topographies instead.

### 3.10 Total Distinct General Topographies Definition

We defined the total distinct general topographies as the sum of distinct general topographies across all patients. For example, if all 55 patients presented with three distinct topographies, the total distinct general topographies would be 165 (55×3).

### 3.11 Epicardial Exit Site

The epicardium is the region most distant from the conduction system. Consequently, ventricular arrhythmias with an exit site in this location exhibit distinctive characteristics. We considered a PVC to be epicardial if it met any of the following three criteria: a QRS duration equal to or greater than 200 ms; the presence of a pseudo‐delta wave lasting more than 34 ms; or a maximum deflection index equal to or greater than 0.55. The maximum deflection index is defined as the interval from the earliest ventricular activation to the earliest maximum deflection of the precordial leads [10].

### 3.12 Data Collection

This is an ambidirectional cohort study. Retrospective data were obtained from patients' records, while prospective data were collected during a 12‐month follow‐up period after enrollment.

### 3.13 Device Therapy Protocol

The detection rate for the VT‐1 zone was programmed between 120 and 180 bpm, depending on the patient. For VT‐2, the range was set between 180 and 200 bpm, and for VF, it was programmed at greater than 200 bpm. The detection counting criteria were configured as follows: 40 beats for VT‐1, 20 beats for VT‐2, and 18 out of 24 beats for VF. At our center, no therapies were programmed in the VT‐1 zone. For the VT‐2 zone, two distinct ATP interventions were typically programmed before shock delivery, each consisting of five attempts with ten pulses per attempt. The first five attempts were delivered at 88% or 85% of the tachycardia cycle length (TCL), depending on the manufacturer and device model, while the final five attempts were set at 75% of the TCL. In the VF zone, a single ATP attempt was always programmed before shock delivery. Shocks were delivered at a minimum of 20 J, up to a maximum of 40 J. In specific scenarios, such as cases involving channelopathies, no ATP was programmed, and the device was set to proceed directly to shocks upon detection of an arrhythmia.

### 3.14 Statistical Analysis

For descriptive statistics, we employed counts, percentages, medians, means, and standard deviations, as applicable. Normality was assessed using the Shapiro–Wilk test. For inferential statistics, we analyzed PVCs according to their axial topography.

Associations between topography and other factors were investigated using generalized mixed models (GMM), with a separate model for each axis. The GMM approach was chosen because it accommodates dependent variables of various types (qualitative or quantitative). This method does not require a normal distribution or homogeneity of variance and is particularly suited for smaller samples. It also accounts for correlated measures and allows for the inclusion of random effects in the model.

Individuals were included as random intercept variables in the model. The set of topographies for each axis was included as the dependent variable, while other variables were treated as independent. The two exams and the PVC events recorded during each exam were included as control variables. A multinomial distribution with a logit link function was assumed. The effect of independent variables on the likelihood of PVC occurrence in each component of each axis was measured by calculating the odds ratio, with 95% confidence intervals computed using the Wald method. Statistical significance was assessed via *z*‐tests, complemented by effect size calculations, where the *z* statistic was converted to the r coefficient.

To analyze the distribution of general topographies with respect to the underlying cardiopathy, Cochran's Q test was employed. Post hoc analyses were conducted using Dunn's test with Bonferroni correction for multiple comparisons. The statistical significance level was set at 5% (*p* ≤ 0.05). In addition to statistical significance, results were also interpreted based on effect size, as *p* values can be significantly influenced by sample size, while effect size offers insight into the practical impact of the study's findings (Supplementary Methods). SPSS Statistics software, version 27.0 (IBM Corp., Armonk, NY, USA), was used for analysis. For generalized mixed models, we used Jamovi software, version 2.4.11 (The jamovi Project, 2023), along with the GAMLj: General Analyses for Linear Models and parameters packages for R, version 4.1.

## 4 Results

### 4.1 Population Characteristics

**FIGURE 3:**
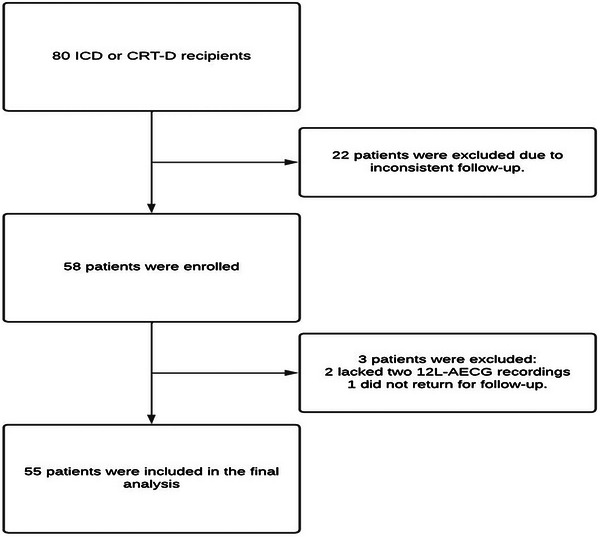
Study flowchart. Out of 80 patients evaluated for inclusion, 22 were excluded due to inconsistent follow‐up. A total of 58 patients were included in the study. Three additional patients were excluded from the final analysis because they either lacked two 12‐lead AECG recordings or failed to return for follow‐up.

During the analysis of our database, 80 patients met the inclusion criteria and were further evaluated. Of these, 22 were excluded from the study, primarily due to inconsistent follow‐up that did not meet the required 12‐month evaluation intervals. We successfully enrolled 58 patients. However, during the follow‐up period, we were unable to obtain 12‐lead ambulatory electrocardiograms from two patients, and one patient did not return for follow‐up after enrollment. Thus, our final sample consisted of 55 patients (Figure 3). The mean age and ejection fraction were 61.9 ± 13.4 years and 49.1 ± 16.1%, respectively. Further details are provided in Table 1.

**TABLE 1:** Population characteristics.

Characteristic	Count (%)
**Gender**	
Male	33 (60.00)
Female	22 (20.00)
**Form of prevention**	
Primary	6 (10.91)
Secondary	49 (89.09)
**Device type**	
ICD	47 (85.54)
CRT‐D	8 (14.46)
**NYHA class**	
I	34 (61.81)
II	18 (32.72)
III	3 (5.46)
**Underlying arrhythmic condition**	
Chagas disease	23 (41.28)
Ischemic heart disease	8 (14.5)
Channelopathies	5 (9.09)
Idiopathic	5 (9.09)
Dilated cardiomyopathy	4 (7.27)
Hypertrophic cardiomyopathy	3 (5.46)
Arrhythmogenic cardiomyopathy	3 (5.46)
Others	4 (7.27)
**Usage of amiodarone**	36 (65.5)
**Usage of beta‐blocker**	49 (89.1)
**Syncope**	16 (29.1)

Abbreviations: CRT‐D, cardiac resynchronization therapy defibrillator; ICD, implantable cardioverter‐defibrillator; NYHA, New York Heart Association.

### 4.2 Follow‐Up Time

After enrollment, each patient was followed for a period of 12 months. As detailed in the methods section, the number of evaluations varied among patients, resulting in three to six prospective evaluations per patient. The mean follow‐up duration was 41.12 ± 13.48 months.

### 4.3 12L‐AECG Results

We collected a total of 110 12L‐AECGs, two per patient. Only four patients exhibited no PVCs on both of their exams. The PVC count for a single exam ranged from 0 to 19,120, with a median of 91.5 (interquartile range 1370). The average QRS duration of PVCs was 164.45 ± 24.80 ms. Patients presented with an average of 1.96 ± 0.96 distinct general topographies. PVCs arising from 18 of the 24 possible general topographies were recorded (Table S8). The total sum of distinct general topographies across all patients was 111. Data is summarized in Table 2.

**TABLE 2:** 12L‐AECG results.

	Count (%)
**Data**	
12L‐AECG exams	110 (100)
Distinct general topographies across all patients	111 (100)
Presence of VT	11 (10)
**Axial topographies distribution**	
**Lateroseptal axis**	
Lateral	31 (27.9)
Central	34 (30.6)
Septal	46 (41.4)
**Anteroinferior axis**	
Anterior	49 (44.1)
Medium	13 (11.7)
Inferior	49 (44.1)
**Apicobasal axis**	
Apical	51 (45.9)
Intermedium	11 (9.9)
Basal	49 (44.1)
**General topographies distribution**	
Septal‐inferior‐apical	23 (20.7)
Central‐anterior‐basal	18 (16.2)
Lateral‐anterior‐basal	15 (13.5)
Central‐inferior‐apical	11 (9.9)
Others	44 (39.7)

Abbreviations: 12L‐AECG, twelve‐lead ambulatory electrocardiogram; VT, ventricular tachycardia.

### 4.4 Device Therapies

During follow‐up, 19 patients received therapies, either antitachycardia pacing (ATP) or shock. The median number of ATP interventions per patient was four, ranging from 1 to 56. In nearly half of the cases, tachycardia was aborted by a single ATP. When one ATP did not terminate the tachycardia, a median of three ATP interventions were required to resolve the episode. When ICD shocks were necessary, they successfully terminated tachycardia in more than 94% of cases on the first attempt. Only three patients required more than one shock during a single episode of ventricular tachycardia (VT). The highest number of shocks needed to terminate an episode of tachycardia was 11, which occurred in a patient with idiopathic ventricular fibrillation while swimming. Four patients experienced inappropriate therapies, all due to supraventricular tachycardia being misinterpreted by the device as VT. Further details are summarized in Table 3.

**TABLE 3:** Devices therapies.

Therapies or results	Count (percentage)
Patients with therapies	19/55 (34.54)
Patients with inappropriate therapies	4/19 (21.05)
Number of patients who received ATP	19
**Total ATP events**	**192**
Successful reversion on the first ATP	92 (47.91)
Reversions after two or more ATP	44 (22.91)
Maximum number of ATP for reversion	6
**Number of shock events**	**57**
Successful reversion on the first shock	54 (94.73)
Maximum number of shocks before reversion	11
Number of patients who received shocks	12

Abbreviations: ATP, antitachycardia pacing; ICD, Implantable cardioverter‐defibrillator.

### 4.5 General Topography Distribution

We identified statistically significant differences in the distribution of general topographies across five cardiopathies: hypertrophic cardiomyopathy (*p* = 0.038), idiopathic dilated cardiomyopathy (*p* = 0.005), Chagas disease (*p* < 0.001), ischemic heart disease (*p* < 0.001), and channelopathies (*p* = 0.025). However, in the post hoc analysis, it was not possible to determine which specific topographies accounted for these differences, with the exception of Chagas disease. Among patients with Chagas disease, PVCs arising from the septal‐inferior‐apical (*p* ≤ 0.004), central‐anterior‐basal (*p* ≤ 0.004), and lateral‐anterior‐basal (*p* < 0.001) topographies were more frequent than the others. Among these three topographies, no single one was significantly more prevalent than the others (*p* > 0.999).

### 4.6 Axial Topography Analysis

We found no significant differences in axial topography distribution between genders or between patients presenting with syncope on the 12L‐AECG (Tables S3 and S4). Additionally, no axial topography was correlated with the number of ATP events, VT terminations by the first ATP, or the number of shocks (Tables S5–S7). However, the following parameters were correlated with specific axial topographies.

### 4.7 Therapies

Patients who received therapies, either ATP or shocks, exhibited a different distribution of PVCs along the apicobasal axis. If a patient received at least one therapy during follow‐up, they had a 2.86‐fold increased likelihood of presenting with PVCs with exit sites in the intermedium segment of the apicobasal axis compared to the basal segment. Additionally, there was a 1.66‐fold increased risk of a basal PVC compared to an apical PVC. The most significant increase was observed in the intermedium segment compared to the apical segment, with a 4.78‐fold increase. In other words, PVCs resulting from an exit site in the intermedium segment of the apicobasal axis were associated with a higher risk of requiring therapies. Further details are summarized in Table 4.

**TABLE 4:** Therapy risk distribution according to PVC topographies.

Axis	Comparison	Odds Ratio	95% CI	*z*	*p*	*r*
Latero septal	Lateral vs. central	0.82	0.32−2.10	−0.404	0.686	0.035
	Lateral vs. septal	0.66	0.27−1.62	−0.906	0.365	0.079
	Central vs. septal	0.80	0.34−1.92	−0.496	0.620	0.043
Antero inferior	Anterior vs. medium	1.57	0.48−5.14	0.738	0.461	0.064
	Anterior vs. inferior	1.15	0.54−2.45	0.354	0.723	0.031
	Medium vs. inferior	0.73	0.22−2.42	−0.509	0.610	0.044
Apico basal	Apical vs. intermedium	4.78	1.19−19.26	2.201	**0.028** ^*^	0.191^†^
	Apical vs. basal	1.66	0.75−3.66	1.248	0.212	0.108^†^
	Intermedium vs. basal	2.86	0.71−11.11	−1.488	0.137	0.129^†^

Abbreviation: 95% CI: 95% confidence interval.

* statistically significant value (*p* ≤ 0.05).

^†^ small effect size.

### 4.8 NYHA Class

PVCs exit sites in the septal portion of the lateroseptal axis were associated with higher NYHA classes. Each increment in NYHA class resulted in a 2.22‐fold higher risk of presenting with a septal PVC compared to a central PVC, and a 1.71‐fold higher risk compared to a lateral PVC. Patients with higher NYHA classes more frequently presented with septal PVCs (Table S1). The central illustration depicts the relationship between NYHA classes, the presence of therapies, and PVC topography.

### 4.9 QRS Duration

For each additional millisecond of QRS duration, there was a 1.04‐fold increase in the odds of a lateral exit site compared to a central one, and a 1.03‐fold increase in the likelihood of a septal exit site compared to a central one. In other words, QRS duration was not evenly distributed along the lateroseptal axis, with central PVCs exhibiting narrower QRS durations compared to other topographies of the lateroseptal axis (Table S2).

### 4.10 Epicardial PVCs

A total of eight patients presented with PVCs whose characteristics were consistent with an epicardial exit site. Four had Chagas disease, two had ischemic heart disease, one had idiopathic dilated cardiomyopathy, and one had congenital heart disease. These patients did not differ significantly from the others in terms of PVC count (*p* = 0.966), age (*p* = 0.777), NYHA class (*p* = 0.257), or presence of ICD therapies (*p* = 0.849). However, they had a significantly lower ejection fraction compared to the other patients (median: 31.5; interquartile range: 20.5 vs. median: 55; interquartile range: 21; *p* = 0.008).

## 5 Discussion

To our knowledge, this is the first study to investigate the correlation between the exit site of PVCs and their impact on the prognosis and clinical features of ICD recipients. We identified a higher risk of device therapies associated with PVCs arising from the intermedium region of the apicobasal axis. We hypothesize that this occurs because this region is centrally located in the left ventricle. The intermedium region occupies a strategic position, relatively close to all areas of the LV. Proximity to reentrant circuits is a well‐established factor for the induction of arrhythmias [11, 12]. We believe that the short distance to scars across various regions of the heart may render intermedium PVCs more likely to induce VT. Consequently, these PVCs more frequently result in device therapies compared to those from other regions.

The topography of PVCs had no impact on ATP success rate. We found ATP to be effective in 70.82% of VTs, a success rate comparable to that reported in the seminal PAINFREE trial [13]. This is an interesting finding given the differences between the populations of the two studies. Our cohort consisted mainly of patients with Chagas cardiomyopathy, while the PAINFREE trial primarily enrolled ischemic patients. Although some investigators have evaluated the efficacy of ICDs in Chagas cardiomyopathy [14, 15, 16], they did not report ATP success rates. Our findings may suggest that ATP is highly effective in Chagas cardiomyopathy, and upcoming trials may provide further evidence to support this hypothesis.

PVCs arising from the central part of the lateroseptal axis had shorter QRS durations compared to those arising from the septal or lateral portions. It is generally expected that QRS duration increases as the PVC exit site moves toward the lateral wall, due to ventricular activation occurring in series rather than in parallel, as is the case with central and septal PVCs. This explanation applies to structurally normal hearts, but in structurally diseased hearts, such as those with fibrosis, conduction can become anisotropic. The extent of fibrosis varies from patient to patient, and in Chagas cardiomyopathy, this variability may have contributed to this counterintuitive result. However, this study alone cannot fully explain this phenomenon. Previous publications have examined the impact of fibrosis on QRS duration [17, 18], but they were limited to quantifying fibrosis and did not analyze its distribution across the left ventricle. Further investigation into how the location of fibrosis influences QRS duration would be needed to confirm our findings.

Patients with septal PVCs were more symptomatic, as indicated by higher NYHA classes. To our knowledge, the relationship between the PVC exit site and NYHA class has not yet been thoroughly investigated. One study [19] suggested that PVC morphology and origin had no effect on the normalization of clinical symptoms after ablation. However, that study only enrolled six patients, which may have limited its power to support or refute any conclusions. It has been well‐established in the literature that apical ventricular pacing has detrimental effects on ventricular function [20], and apical pacing is closely related to the interventricular septum. Although our study did not specifically evaluate topographies in pairwise combinations, we speculate that PVCs arising from the apical portion of the septum may be responsible for the association with higher NYHA classes. Visually, the central illustration suggests a tendency for higher NYHA classes to be associated with the apical region of the interventricular septum. However, given the distribution of general topographies in our cohort, we did not have sufficient statistical power to test this hypothesis.

Some cardiopathies exhibit a predilection for specific anatomical regions of the heart. In patients with Chagas disease, we identified three topographies that were significantly more prevalent. These sites, located in the apical and basal regions, align with previous reports in the literature describing the most frequently affected areas in Chagas cardiomyopathy [21]. Four additional cardiopathies also demonstrated significant differences in their topographic distribution. However, the post hoc analysis was unable to accurately determine which topographies accounted for these differences, likely due to the relatively small number of patients in each group.

Epicardial PVCs were associated with lower ejection fractions. A previous animal study [22] demonstrated that pacing from the left ventricular epicardium led to more pronounced cardiomyopathy, characterized by greater dyssynchrony, lower stroke volumes, and reduced contractility (as measured by the first‐order derivative of pressure with respect to time, dP/dt). Our results are consistent with these previously reported findings. However, this association does not imply causality and should be interpreted with caution. An alternative explanation could be that patients developed epicardial ectopic beats because they had more extensive transmural fibrosis, and therefore, their hearts were intrinsically less contractile than those of the other patients.

## 6 Limitations

Algorithms for predicting the origin of PVCs primarily describe arrhythmias in structurally normal hearts. Predicting the origin of scar‐related PVCs and VTs is significantly more challenging. A single scar may have multiple exit sites, and the same focus, capturing the ventricle through different exits, can produce entirely different QRS complexes. Consequently, the few algorithms available for evaluating scar‐related ventricular arrhythmias have limited sensitivity and specificity and are applicable only in specific scenarios [23].

Our population was highly heterogeneous with respect to underlying cardiac pathology. This makes the task of locating the exact exit site of the PVC very challenging and, in some cases, may have resulted in the misclassification of certain PVC topographies. A more homogeneous group would have improved the study's internal validity. However, it would have been detrimental to its external validity, as not all patients receiving an ICD, for example, have myocardial scarring. We aimed to make our analysis as broadly applicable as possible.

We were unable to thoroughly analyze the influence of PVC general topography on patient prognosis due to the low incidence of certain topographies. Some topographies were recorded only once, while others were not detected at all.

A sample size of 55 was insufficient to conduct a robust analysis of the 24 possible general topographies. A larger sample size would be required for this purpose. The major limitation of our study was the sample size. Although we enrolled every eligible patient from our institution, many had to be excluded from the final analysis due to irregular follow‐up. The limited sample size did not allow for a reliable assessment of whether specific topographic distributions were more common among each cardiopathy. This limitation may have introduced bias into the analysis.

Most of our patients received their implants for secondary prevention, which may have introduced bias into our analysis and limited the generalizability of our findings to populations with a higher proportion of patients receiving implants for primary prevention.

Our objective was to evaluate how the three‐dimensional distribution of PVCs impacted the prognosis of ICD recipients. Given this aim, we did not collect data on most traditional Holter parameters, such as coupling intervals, presence of couplets and triplets, ST deviations, and others. Although we could have collected such data, these parameters have already been extensively studied and would not have contributed novel insights.

Finally, our data would have been more precise had we employed electroanatomic mapping in our study. However, subjecting patients to electroanatomic mapping solely to verify the exit site of their PVCs, without any other clear indication, would have been unethical. The potential risks of the procedure, without tangible benefits for the patient, are not justifiable.

## 7 Clinical Perspectives

Our study is the first to demonstrate that the spatial distribution of PVCs affects the prognosis of ICD recipients. Future studies are needed to draw definitive conclusions. However, if our findings are confirmed in larger studies, this may influence the management of these patients. For instance, septal PVCs could become a marker of an unfavorable heart failure prognosis, while intermedium PVCs may indicate the need for more intensive antiarrhythmic treatment, as these patients are more likely to require device therapies.

## 8 Conclusion

Different PVC topographies influence the prognosis of ICD recipients in distinct ways. Further research is necessary to elucidate the relationship between PVC location and the clinical outcomes of ICD recipients. If validated in future studies, our findings could have significant implications not only for prognosis but also for the treatment strategies employed for ICD recipients.

## Author Contributions

**Carlos Arthur Hansel Diniz da Costa**: conceptualization, methodology, investigation, formal analysis, writing – original draft. **Gabriela Menichelli Medeiros Coelho**: investigation, visualization. **Rhanniel Theodorus Helhyas Oliveira Shilva Gomes Villar**: investigation, visualization. **Gabriela Rodrigues de Oliveira**: investigation, visualization. **Pedro Henrique Correia Filgueiras**: investigation, visualization. **Enia Lúcia Coutinho**: investigation, visualization. **Claudio Cirenza**: formal analysis, writing – reviewing, and editing. **Angelo Amato Vincenzo de Paola**: conceptualization, formal analysis, writing – reviewing, and editing.

## Ethics Statement

This study was approved by the Ethics Committee of the Federal University of São Paulo (CAAE: 64353722.5.0000.5505).

## Consent

Informed consent was obtained from all participants.

## Conflicts of Interest

The authors declare no conflicts of interest.

## Supporting information




**Table S1**: NYHA class risk distribution according to PVC topographies
**Table S2**: Longer QRS duration risk distribution according PVC topographies
**Table S3**: Gender distribution according to PVC topographies
**Table S4**: Syncope risk distribution according to PVC topographies
**Table S5**: ATP risk distribution according to PVC topographies
**Table S6**: Reversions on the first ATP distribution according to PVC topographies
**Table S7**: Shock risk distribution according to PVC topographies
**Table S8**: PVC general topography distribution
**Figure S1**: Twelve‐lead ambulatory electrocardiogram

## Data Availability

The data underlying this article will be shared on reasonable request to the corresponding author.
