# Supplementary material for: The Impact of the Spatial Distribution of Ventricular Extrasystoles on Implantable Cardioverter‐Defibrillator Recipients
Source: Pacing Clin Electrophysiol. 2025 Aug 25;48(10):1157–66. doi: 10.1111/pace.70033 (PMC12504925; doi:10.1111/pace.70033)
Supplement: Supplementary file 1 — Table S1: NYHA class risk distribution according to PVC topographies Table S2: Longer QRS duration risk distribution according PVC topographies Table S3: Gender distribution according to PVC topographies Table S4: Syncope risk distribution according to PVC topographies Table S5: ATP risk distribution according to PVC topographies Table S6: Reversions on the first ATP distribution according to PVC topographies Table S7: Shock risk distribution according to PVC topographies Table S8: PVC general topography distribution Figure S1: Twelve‐lead ambulatory electrocardiogram [file PACE-48-1157-s001.docx]

**Supplementary Material**

This supplementary material has been elaborated by authors to provide additional information about our research.

Supplement to: The Impact of the Spatial Distribution of Ventricular Extrasystoles on Implantable Cardioverter-Defibrillator Recipients.

**The Impact of the Spatial Distribution of Ventricular Extrasystoles on Implantable Cardioverter-Defibrillator Recipients**

**SUPPLEMENTARY MATERIALS**

| **TABLE OF CONTENTS** | **PAGE** |
| --- | --- |
|  |  |
| **1. SUPPLEMENTARY METHODS** | **2** |
| Effect size interpretation | 2 |
| **2. SUPPLEMENTARY TABLES** | **3** |
| Table S1. NYHA class risk distribution according to PVC topographies | 3 |
| Table S2. Longer QRS duration risk distribution according PVC topographies | 3 |
| Table S3. Gender distribution according to PVC topographies | 3 |
| Table S4. Syncope risk distribution according to PVC topographies | 4 |
| Table S5. ATP risk distribution according to PVC topographies | 4 |
| Table S6 Reversions on the first ATP distribution according to PVC topographies | 4 |
| Table S7. Shock risk distribution according to PVC topographies | 5 |
| Table S8. PVC general topography distribution: | 5 |
| **3. SUPPLEMENTARY IMAGES** | **6** |
| Figure S1. Twelve-lead ambulatory electrocardiogram | 6 |
| **4. REFERENCES** | **7** |

**1. SUPPLEMENTARY METHODS**

**Effect size interpretation**

For the interpretation of effect sizes, we employed the classification proposed by Cohen [1]. Specifically, for the *r* coefficient and correlation coefficients, we adopted the following criteria:

- Small: between |0.100| and |0.299|.
- Medium: between |0.300| and |0.499|.
- Large: greater than |0.500|.

**2. SUPPLEMENTARY TABLES**

**Table S1. NYHA class risk distribution according to PVC topographies**

| **Axis** | **Comparison** | ***Odds Ratio*** | **95% CI** | **z** | **p** | **r** |
| --- | --- | --- | --- | --- | --- | --- |
| Latero  septal | Lateral vs Central | 0.77 | 0.33 – 1.78 | -0.610 | 0.542 | 0.053 |
|  | Lateral vs Septal | 1.71 | 0.83 – 3.55 | 1.450 | 0.147 | 0.126^†^ |
|  | Central vs Septal | 2.22 | 1.08 – 4.55 | 2.172 | **0.030*** | 0.188^†^ |
| Antero  inferior | Anterior vs Medium | 0.52 | 0.17 – 1.60 | -1.144 | 0.253 | 0.099 |
|  | Anterior vs Inferior | 0.98 | 0.54 – 1.76 | -0.074 | 0.941 | 0.006 |
|  | Medium vs Inferior | 1.89 | 0.61 – 5.88 | 1.098 | 0.272 | 0.095 |
| Apico  basal | Apical vs Intermedium | 0.98 | 0.32 – 2.96 | -0.036 | 0.971 | 0.003 |
|  | Apical vs Basal | 1.15 | 0.64 – 2.01 | 0.469 | 0.639 | 0.041 |
|  | Intermedium vs Basal | 1.17 | 0.38 – 3.57 | 0.278 | 0.781 | 0.024 |

95% CI: 95% Confidence interval; *: statistically significant value (p ≤ 0,05); ^†^: small effect size.

**Table S2. Longer QRS duration risk distribution according PVC topographies**

| **Axis** | **Comparison** | ***Odds Ratio*** | **95% CI** | **z** | **p** | **r** |
| --- | --- | --- | --- | --- | --- | --- |
| Latero  septal | Lateral vs Central | 0.96 | 0.94 – 0.98 | -3.532 | **< 0.001*** | 0.306^††^ |
|  | Lateral vs Septal | 0.99 | 0.97 – 1.01 | -1.398 | 0.162 | 0.121 |
|  | Central vs Septal | 1.03 | 1.01 – 1.05 | 2.624 | **0.009*** | 0.228^†^ |
| Antero  inferior | Anterior vs Medium | 0.97 | 0.95 – 1.00 | -1.889 | 0.059 | 0.164^†^ |
|  | Anterior vs Inferior | 1.00 | 0.98 – 1.01 | -0.336 | 0.737 | 0.029 |
|  | Medium vs Inferior | 1.03 | 1.00 – 1.05 | 1.708 | 0.088 | 0.148^†^ |
| Apico  basal | Apical vs Intermedium | 1.00 | 0.98 – 1.03 | 0.107 | 0.915 | 0.009 |
|  | Apical vs Basal | 1.01 | 0.99 – 1.02 | 0.393 | 0.694 | 0.034 |
|  | Intermedium vs Basal | 1.00 | 0.98 – 1.03 | 0.076 | 0.939 | 0.007 |

95% CI: 95% Confidence interval; *: statistically significant value (p ≤ 0,05); ^†^: small effect size;

^††^: medium effect size.

**Table S3. Gender distribution according to PVC topographies**

| **Axis** | **Comparison** | ***Odds Ratio*** | **95% CI** | **z** | **p** | **r** |
| --- | --- | --- | --- | --- | --- | --- |
| Latero  septal | Lateral vs Central | 1.10 | 0.44 – 2.76 | 0.196 | 0.844 | 0.017 |
|  | Lateral vs Septal | 1.00 | 0.42 – 2.40 | 0.008 | 0.993 | 0.001 |
|  | Central vs Septal | 0.92 | 0.40 – 2.13 | -0.201 | 0.841 | 0.017 |
| Antero  inferior | Anterior vs Medium | 1.83 | 0.52 – 6.51 | 0.937 | 0.349 | 0.081 |
|  | Anterior vs Inferior | 1.15 | 0.55 – 2.39 | 0.363 | 0.717 | 0.031 |
|  | Médio vs Medium | 0.63 | 0.17 – 2.25 | -0.721 | 0.471 | 0.063 |
| Apico  basal | Apical vs Intermedium | 1.17 | 0.30 – 4.61 | 0.226 | 0.821 | 0.020 |
|  | Apical vs Basal | 0.91 | 0.43 – 1.92 | -0.255 | 0.799 | 0.022 |
|  | Intermedium vs Basal | 0.78 | 0.20 – 3.07 | -0.360 | 0.719 | 0.031 |

95% CI: 95% Confidence interval.

**Table S4. Syncope risk distribution according to PVC topographies**

| **Axis** | **Comparison** | ***Odds Ratio*** | **95% CI** | **z** | **p** | **r** |
| --- | --- | --- | --- | --- | --- | --- |
| Latero  septal | Lateral vs Central | 1.77 | 0.63 – 4.96 | 1.084 | 0.278 | 0.094 |
|  | Lateral vs Septal | 0.76 | 0.27 – 2.15 | -0.515 | 0.606 | 0.045 |
|  | Central vs Septal | 0.44 | 0.17 – 1.11 | -1.745 | 0.081 | 0.151^†^ |
| Antero  inferior | Anterior vs Medium | 0.72 | 0.18 – 2.93 | -0.456 | 0.648 | 0.040 |
|  | Anterior vs Inferior | 1.15 | 0.52 – 2.55 | 0.345 | 0.730 | 0.030 |
|  | Medium vs Inferior | 1.59 | 0.39 – 6.47 | 0.651 | 0.515 | 0.056 |
| Apico  basal | Apical vs Intermedium | 1.57 | 0.34 – 7.23 | 0.583 | 0.560 | 0.051 |
|  | Apical vs Basal | 2.28 | 0.96 – 5.45 | 1.855 | 0.064 | 0.161^†^ |
|  | Intermedium vs Basal | 1.44 | 0.33 – 6.24 | 0.483 | 0.629 | 0.042 |

95% CI: 95% Confidence interval; ^†^: small effect size.

**Table S5. ATP risk distribution according to PVC topographies**

| **Axis** | **Comparison** | ***Odds Ratio*** | | **95% CI** | **z** | **p** | **r** |
| --- | --- | --- | --- | --- | --- | --- | --- |
| Latero  septal | Lateral vs Central | | 1.06 | 0.97 – 1.15 | 1.193 | 0.233 | 0.174^†^ |
|  | Lateral vs Septal | | 1.05 | 0.97 – 1.15 | 1.169 | 0.242 | 0.171^†^ |
|  | Central vs Septal | | 1.00 | 0.95 – 1.04 | -0.140 | 0.889 | 0.020 |
| Antero  inferior | Anterior vs Medium | | 1.02 | 0.95 – 1.09 | 0.522 | 0.602 | 0.076 |
|  | Anterior vs Inferior | | 1.02 | 0.97 – 1.07 | 0.759 | 0.448 | 0.111^†^ |
|  | Medium vs Inferior | | 1.00 | 0.93 – 1.08 | 0.095 | 0.924 | 0.014 |
| Apico  basal | Apical vs Intermedium | | 0.97 | 0.90 – 1.05 | -0.841 | 0.400 | 0.123^†^ |
|  | Apical vs Basal | | 0.98 | 0.94 – 1.03 | -0.721 | 0.471 | 0.105^†^ |
|  | Intermedium vs Basal | | 1.02 | 0.94 – 1.10 | 0.393 | 0.695 | 0.057 |

95% CI: 95% Confidence interval; ^†^: small effect size.

**Table S6 Reversions on the first ATP distribution according to PVC topographies**

| **Axis** | **Comparison** | ***Odds Ratio*** | **95% CI** | **z** | **p** | **r** |
| --- | --- | --- | --- | --- | --- | --- |
| Latero  septal | Lateral vs Central | 1.06 | 0.94 – 1.18 | 0.952 | 0.341 | 0.139^†^ |
|  | Lateral vs Septal | 1.06 | 0.95 – 1.18 | 0.981 | 0.327 | 0.143^†^ |
|  | Central vs Septal | 1.00 | 0.93 – 1.08 | 0.014 | 0.989 | 0.002 |
| Antero  inferior | Anterior vs Medium | 1.05 | 0.96 – 1.15 | 1.076 | 0.282 | 0.157^†^ |
|  | Anterior vs Inferior | 1.01 | 0.93 – 1.09 | 0.149 | 0.881 | 0.022 |
|  | Medium vs Inferior | 0.96 | 0.86 – 1.07 | -0.803 | 0.422 | 0.117^†^ |
| Apico  basal | Apical vs Intermedium | 0.96 | 0.85 – 1.08 | -0.710 | 0.478 | 0.104^†^ |
|  | Apical vs Basal | 0.99 | 0.92 – 1.08 | -0.144 | 0.886 | 0.021 |
|  | Intermedium vs Basal | 1.04 | 0.92 – 1.17 | 0.601 | 0.548 | 0.088 |

95% CI: 95% Confidence interval; ^†^: small effect size.

**Table S7. Shock risk distribution according to PVC topographies**

| **Axis** | **Comparison** | ***Odds Ratio*** | **95% CI** | **z** | **p** | **r** |
| --- | --- | --- | --- | --- | --- | --- |
| Latero  septal | Lateral vs Central | 1.00 | 0.85 – 1.17 | -0.017 | 0.986 | 0.003 |
|  | Lateral vs Septal | 0.82 | 0.59 – 1.15 | -1.160 | 0.246 | 0.215^†^ |
|  | Central vs Septal | 0.83 | 0.61 – 1.12 | -1.244 | 0.214 | 0.231^†^ |
| Antero  inferior | Anterior vs Medium | 0.88 | 0.64 – 1.21 | -0.771 | 0.441 | 0.143^†^ |
|  | Anterior vs Inferior | 0.95 | 0.85 – 1.06 | -0.998 | 0.319 | 0.185^†^ |
|  | Medium vs Inferior | 1.07 | 0.78 – 1.47 | 0.424 | 0.672 | 0.079 |
| Apico  basal | Apical vs Intermedium | 0.71 | 0.35 – 1.42 | -0.972 | 0.331 | 0.180^†^ |
|  | Apical vs Basal | 1.02 | 0.91 – 1.14 | 0.338 | 0.735 | 0.063 |
|  | Intermedium vs Basal | 1.44 | 0.72 – 2.90 | 1.024 | 0.306 | 0.190^†^ |

95% CI: 95% Confidence interval; ^†^: small effect size.

**Table S8. PVC general topography distribution:**

| **General Topography** | **Counts** | **Percentage** |
| --- | --- | --- |
| Septal-Anterior-Basal | 6 | 5.4 % |
| Septal-Medium-Apical | 4 | 3.6 % |
| Septal-Medium-Intermedium | 3 | 2.7 % |
| Septal-Medium-Basal | 7 | 6.3 % |
| Septal-Inferior-Apical | 23 | 20.7 % |
| Septal-Inferior-Intermedium | 1 | 0.9 % |
| Septal-Inferior-Basal | 2 | 1.8 % |
| Central-Anterior-Apical | 3 | 2.7 % |
| Central-Anterior-Basal | 18 | 16.2 % |
| Central-Inferior-Apical | 11 | 9.9 % |
| Central-Inferior-Intermedium | 1 | 0.9 % |
| Central-Inferior-Basal | 1 | 0.9 % |
| Lateral-Anterior-Apical | 4 | 3.6 % |
| Lateral-Anterior-Intermedium | 2 | 1.8 % |
| Lateral-Anterior-Basal | 15 | 13.5 % |
| Lateral-Inferior-Apical | 6 | 5.4 % |
| Lateral-Inferior-Intermedium | 3 | 2.7 % |
| Lateral-Inferior-Basal | 1 | 0.9 % |

**3. SUPPLEMENTARY IMAGES**

**Figure S1. Twelve-lead ambulatory electrocardiogram**

**
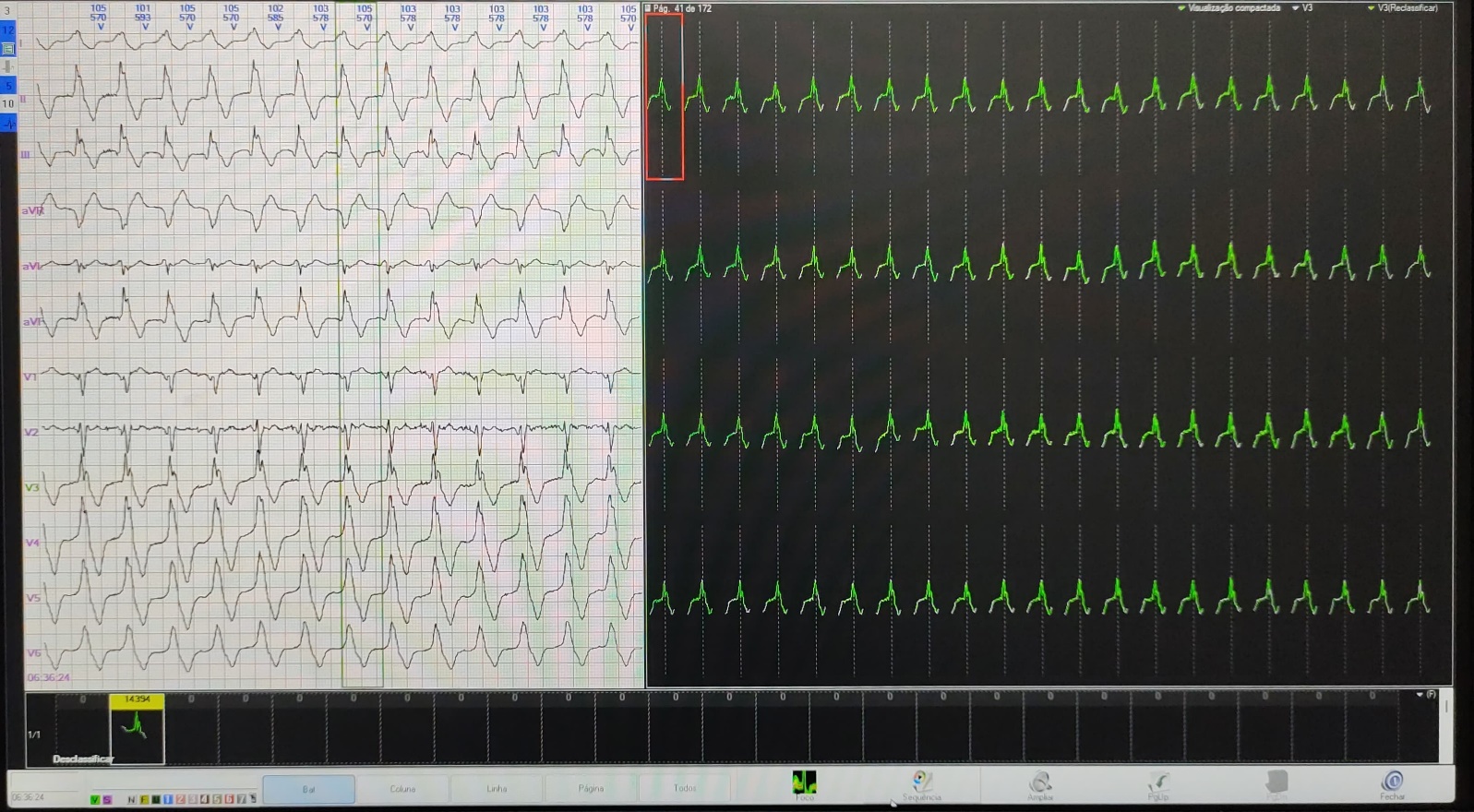
**

Twelve-lead ambulatory electrocardiogram. It consists of the twelve leads of the standard electrocardiogram recorded over a twenty-four hour period.

**4. REFERENCES**

[1] Cohen J. Statistical Power analysis. Current Directions in Psychological Science [Internet]. 1992 Jun 1;1(3):98–101. Available from: https://doi.org/10.1111/1467-8721.ep10768783
